# Evolving role of point-of-care ultrasound in prehospital emergency care: a narrative review

**DOI:** 10.1186/s13049-025-01443-x

**Published:** 2025-07-14

**Authors:** Katharina E. M. Hellenthal, Christian Porschen, Jan Wnent, Matthias Lange

**Affiliations:** 1 Department of Anesthesiology, Intensive Care and Pain Medicine, University Hospital Muenster, Albert-Schweitzer-Campus 1, A1, 48149 Muenster, Germany; 2 Institute for Emergency Medicine, University Hospital Schleswig-Holstein, Kiel, Germany; 3 Department of Anesthesiology and Intensive Care Medicine, University Hospital Schleswig-Holstein, Kiel, Germany

**Keywords:** Prehospital, Point-of-care ultrasound, Respiratory distress, Trauma, Cardiac arrest, Shock

## Abstract

Point-of-care ultrasound is an emerging technology in prehospital emergency care, covering a wide range of medical and traumatic disease patterns. As an ad-hoc imaging modality, it is performed on-scene and during ground or aeromedical transport, enabling experienced prehospital clinicians to diagnose or rule out potentially life-threatening conditions. Rapid ultrasound assessment modulates treatment decisions and guides the choice of transport mode and appropriate hospital destination. In this narrative review, we explore the diagnostic and therapeutic utility of point-of-care ultrasound in key prehospital symptom complexes, including respiratory distress, trauma, cardiac arrest and nontraumatic shock. We provide a concise overview of relevant protocols and ultrasound findings that support the management of prehospital disease patterns and highlight both the benefits and challenges of on-scene ultrasound. In addition, we discuss potential future applications in the context of artificial intelligence. We advocate for large scale clinical trials and underscore the need for comprehensive educational programs focused at skill aquisiton and maintenance, both of which are essential for advancing prehospital emergency care and upholding high standards of quality.

## Background

Within the past several years, due to the technical advances in portable ultrasound machines, prehospital point-of-care ultrasound (PHUS) has become an emerging technology in the management of medical and traumatic disease patterns [1–3]. While ultrasound can present challenges in the prehospital setting, due to ambient light changes, weather conditions, vibrations, limited workspace or experience, it may help the well-trained practitioner in rapid diagnosis and therapy. The versatility of ultrasound enables a wide range of prehospital applications, particularly by guiding initial treatment decisions and supporting the selection of appropriate transport mode and hospital destination [4]. The widespread use of point-of-care ultrasound has led to its implementation in the medical school curriculum, emergency physician training and paramedic training programs [5, 6]. Currently available portable devices are reported to have an accuracy of 90% in emergencies compared to high-end devices, used in hospital settings, offering accessibility for clinicians in the prehospital setting [7]. In the present review, we aim to dissect the evidence regarding the diagnostic value and utility of PHUS and its potential therapeutic benefit in major prehospital symptom complexes. We will guide readers through various protocols and ultrasound findings that aid in the prehospital management of respiratory distress, cardiac arrest, trauma and shock. Furthermore, we highlight their benefits and limitations and outline future perspectives for the interpretation of prehospital ultrasound.

### Point-of-care ultrasound — an emerging technology in prehospital care

The first clinical ultrasound images were developed in the 1940s. Gohe and Wedekind first described the capability of ultrasound to detect tumors, exsudates and abscesses, while the Austrian neurologists Karl and Riederick Dussik attempted to image cerebral ventricules [8]. Later in the 1950s, Donald and Brown used ultrasound for obstetric sonography and to detect abdominal tumors [9]. In 1971, sonography was first used to evaluate blunt abdominal trauma and echocardiography was successfully implemented into emergency settings thereafter [8, 10]. Decades later, the first pocket ultrasound device was launched in 2007 [8]. Approximately 20 years before that, in the Armenian earthquake of 1988, physicians equipped with two ultrasound machines were able to triage 400 blunt trauma patients within two days, thereby opening an era of on-scene ultrasound [11]. Traditionally, in rural or military areas with limited diagnostic equipment, point-of-care ultrasound provides a significant benefit that can alter therapeutic decisions and patient disposition, especially as transport remains challenging [12]. In 2011, PHUS was declared as one of the five most important prehospital research areas [13].

To date, PHUS is an ad-hoc imaging modality not only performed by emergency physicians, but also by paramedics [14–16]. To our knowledge, there is no minimum number of scans performed to be considered as competency for paramedics. For emergency physicians, the American College of Emergency Physicians recommends 25 to 50 scans as indicative of competency, and the European Society for Emergency Medicine is currently developing a special curriculum for emergency point-of-care ultrasound [17]. The German Society for Ultrasound in Medicine (DEGUM) has recently issued a certificate in emergency ultrasound, in which candidates must perform a minimum of 25 supervised focused assessment with sonography for trauma (FAST) exams and 80 focused transthoracic echocardiography exams [18]. For FAST, the initial learning curve is steep and starts to flatten after 30–100 scans [19]. Different studies demonstrated that short training periods lead to successful implementation into prehospital care [14–16]. Even a short four-hour ultrasound training course improved the ability of prehospital physicians to perform ultrasound examinations [20]. In performance of focused transthoracic echocardiography, competency increases after a 10 h course and more than 45 performed scans [21]. Subsequently, there is an important need for structured education programs of skill acquisition and maintenance.

In 2000, the first study assessing the feasibility of ultrasound during helicopter transport found this technique easy to perform among a small group of examiners [22]. In a prospective study assessing the diagnostic value of PHUS in identifying pleural, peritoneal, pericardial effusion and vascular disease (i.e., deep venous thrombosis or arterial flow interruption), ultrasound improved diagnostic accuracy in 67%, decreased diagnostic accuracy in 8% and did not alter diagnosis in 25% of cases. In this study, prehospital diagnosis before and after ultrasonographic examination were expressed in a clinical probability score and compared with the final diagnosis during in-hospital follow-up. The authors themselves speculated that reduced diagnostic accurary in some cases may be explained by technical difficulties such as inadequate patient access, poor examination quality, or limited screen visualization.

Importantly, in patients with uncertain diagnosis, ultrasound improved diagnostic accuracy in 90% of cases (*n* = 115) [23]. Of note, diagnostic accuracy may vary depending on transportation mode. Studies reported diagnostic sensitivity of 95,2% on-scene and of 94,7% during transfer by ground ambulance, while examinations during aeromedical transportation may reduce sensitivity [24, 25]. In this regard, for in-flight extended FAST (eFAST), a sensitivity of 78,6% - in comparison to a computed tomography (CT) scan - was reported [26]. Confounding factors for PHUS including ambient light changes, weather conditions, vibrations, noise and limited workspace present challenges not commonly encountered in the hospital setting. Nevertheless, the potential of PHUS to influence treatment is notable. Ultrasound findings changed treatment in 78% of cases and had additional therapeutic implications in 25% of patients [27, 28]. Still, PHUS may be a double-edged sword in terms of situational awareness. For instance, in a simulated setting, decrease in oxygen saturation during ultrasound performance went unnoticed in 75% of cases. Importantly, only 33% of the participants who missed the desaturation felt confident in using PHUS, while all of those who recognized it (100%) felt confident [29]. Hence, prehospital care providers must possess adequate experience to ensure appropriate application.

Regarding time management, it can be hypothesized that PHUS may extend on-scene time and delay procedural workflows. Conversely, many studies conclude that PHUS does not delay patient management with a reported mean examination time of 2.5 min (1–3 min) [30]. Even echocardiography during out-of-hospital cardiopulmonary resuscitation (CPR) was performed in less than 10 s [27]. In a recent prospective, randomized, multicenter trial, PHUS even significantly reduced time to hospital admission by 13 min and surgery by 15 min suggesting a temporal benefit in addition to diagnostic benefit [31].

Taken together, these data mirror a robust growing body of evidence for widespread implementation of PHUS.

## Methods

For this structured narrative review, a comprehensive literature search of the PubMed database was conducted, combining the search terms “prehospital”, “ultrasound” and the symptom complexes “respiratory distress”, “trauma”, “cardiac arrest” and “shock”. We analyzed respective literature from 1985 to 2025. Additionally, the references of the respective articles were analyzed. Results were thematically categorized and discussed to emphasize key findings and identify future research directions. Key studies have been compiled in Table 1, categorized according to symptom complexes.

**Table 1:** Key studies assessing diagnostic utility of point-of-care ultrasound

**Respiratoy Distress**
Bergmann I, Büttner B, Teut E, et al. Pre-hospital transthoracic echocardiography for early identification of non-ST-elevation myocardial infarction in patients with acute coronary syndrome. *Crit Care*. 2018;22(1):29. 10.1186/s13054-017-1929-1
Jambrik Z, Monti S, Coppola V, et al. Usefulness of ultrasound lung comets as a nonradiologic sign of extravascular lung water. *The American Journal of Cardiology*. 2004;93(10):1265–1270. 10.1016/j.amjcard.2004.02.012
Ketelaars R, Hoogerwerf N, Scheffer GJ. Prehospital Chest Ultrasound by a Dutch Helicopter Emergency Medical Service. *The Journal of Emergency Medicine*. 2013;44(4):811–817. 10.1016/j.jemermed.2012.07.085
Laursen CB, Hänselmann A, Posth S, Mikkelsen S, Videbæk L, Berg H. Prehospital lung ultrasound for the diagnosis of cardiogenic pulmonary oedema: a pilot study. *Scand J Trauma Resusc Emerg Med*. 2016;24:96. Published 2016 Aug 2. 10.1186/s13049-016-0288-2
Lichtenstein D, Goldstein I, Mourgeon E, Cluzel P, Grenier P, Rouby JJ. Comparative Diagnostic Performances of Auscultation, Chest Radiography, and Lung Ultrasonography in Acute Respiratory Distress Syndrome. *Anesthesiology*. 2004;100(1):9–15.
Lichtenstein D. Lung ultrasound in acute respiratory failure an introduction to the BLUE-protocol. *Minerva Anestesiol*. 2009;75(5):313–317
Lyon M, Walton P, Bhalla V, Shiver SA. Ultrasound detection of the sliding lung sign by prehospital critical care providers. *The American Journal of Emergency Medicine*. 2012;30(3):485–488. 10.1016/j.ajem.2011.01.009
Neesse A, Jerrentrup A, Hoffmann S, et al. Prehospital chest emergency sonography trial in Germany: a prospective study. *European Journal of Emergency Medicine*. 2012;19(3):161–166. 10.1097/MEJ.0b013e328349edcc
Prosen G, Klemen P, Strnad M, Grmec Š. Combination of lung ultrasound (a comet-tail sign) and N-terminal pro-brain natriuretic peptide in differentiating acute heart failure from chronic obstructive pulmonary disease and asthma as cause of acute dyspnea in prehospital emergency setting. *Crit Care*. 2011;15(2):R114. 10.1186/cc10140
Schoeneck JH, Coughlin RF, Baloescu C, et al. Paramedic-performed Prehospital Point-of-care Ultrasound for Patients with Undifferentiated Dyspnea: A Pilot Study. *West J Emerg Med*. 2021;22(3):750–755. 10.5811/westjem.2020.12.49254
Staub LJ, Mazzali Biscaro RR, Kaszubowski E, Maurici R. Lung Ultrasound for the Emergency Diagnosis of Pneumonia, Acute Heart Failure, and Exacerbations of Chronic Obstructive Pulmonary Disease/Asthma in Adults: A Systematic Review and Meta-analysis. *J Emerg Med*. 2019;56(1):53–69. 10.1016/j.jemermed.2018.09.009
**Cardiac Arrest**
Breitkreutz R, Price S, Steiger HV, et al. Focused echocardiographic evaluation in life support and peri-resuscitation of emergency patients: A prospective trial. *Resuscitation*. 2010;81(11):1527–1533. 10.1016/j.resuscitation.2010.07.013
Clattenburg EJ, Wroe P, Brown S, et al. Point-of-care ultrasound use in patients with cardiac arrest is associated prolonged cardiopulmonary resuscitation pauses: A prospective cohort study. *Resuscitation*. 2018;122:65–68. 10.1016/j.resuscitation.2017.11.056
Huis In ’T Veld MA, Allison MG, Bostick DS, et al. Ultrasound use during cardiopulmonary resuscitation is associated with delays in chest compressions. *Resuscitation*. 2017;119:95–98. 10.1016/j.resuscitation.2017.07.021
**Hemodynamics**
Keikha M, Salehi-Marzijarani M, Soldoozi Nejat R, Sheikh Motahar Vahedi H, Mirrezaie SM. Diagnostic Accuracy of Rapid Ultrasound in Shock (RUSH) Exam; A Systematic Review and Meta-analysis. *BEAT*. 2018;6(4):271–278. 10.29252/beat-060402
Lichtenstein DA. BLUE-Protocol and FALLS-Protocol. *Chest*. 2015;147(6):1659–1670. 10.1378/chest.14-1313
Perera P, Mailhot T, Riley D, Mandavia D. The RUSH Exam: Rapid Ultrasound in SHock in the Evaluation of the Critically lll. *Emergency Medicine Clinics of North America*. 2010;28(1):29–56. 10.1016/j.emc.2009.09.010
**Trauma**
Canelli R, Leo M, Mizelle J, Shrestha GS, Patel N, Ortega R. Use of eFAST in Patients with Injury to the Thorax or Abdomen. Ingelfinger JR, ed. *N Engl J Med*. 2022;386(10). 10.1056/NEJMvcm2107283
Lin KT, Lin ZY, Huang CC, et al. Prehospital ultrasound scanning for abdominal free fluid detection in trauma patients: a systematic review and meta-analysis. *BMC Emerg Med*. 2024;24(1):7. 10.1186/s12873-023-00919-2
Netherton S, Milenkovic V, Taylor M, Davis PJ. Diagnostic accuracy of eFAST in the trauma patient: a systematic review and meta-analysis. *CJEM*. 2019;21(6):727–738. 10.1017/cem.2019.381
Nunes LW, Simmons S, Hallowell MJ, Kinback R, Trooskin S, Kozar R. Diagnostic performance of trauma US in identifying abdominal or pelvic free fluid and serious abdominal or pelvic injury. *Academic Radiology*. 2001;8(2):128–136. 10.1016/S1076-6332(01)90057-1
Walcher F, Weinlich M, Conrad G, et al. Prehospital ultrasound imaging improves management of abdominal trauma. *Journal of British Surgery*. 2006;93(2):238–242. 10.1002/bjs.5213

## Airway and breathing

### Respiratory distress– diagnostics

#### Lung transthoracic ultrasound

Lung transthoracic ultrasound is a rapid imaging method that facilitates the diagnosis of different etiologies of respiratory distress. Recently, during the COVID-19 pandemic, point-of-care ultrasound enabled healthcare workers to quickly scan and triage thousands of patients raising global awareness of lung ultrasound technology. The most common diagnoses among patients with prehospital respiratory distress include congestive heart failure (16%), pneumonia (15%) and chronic obstructive pulmonary disease (COPD) (13%), all of which ultrasound may help differentiate early [32].

**Fig. 1:**
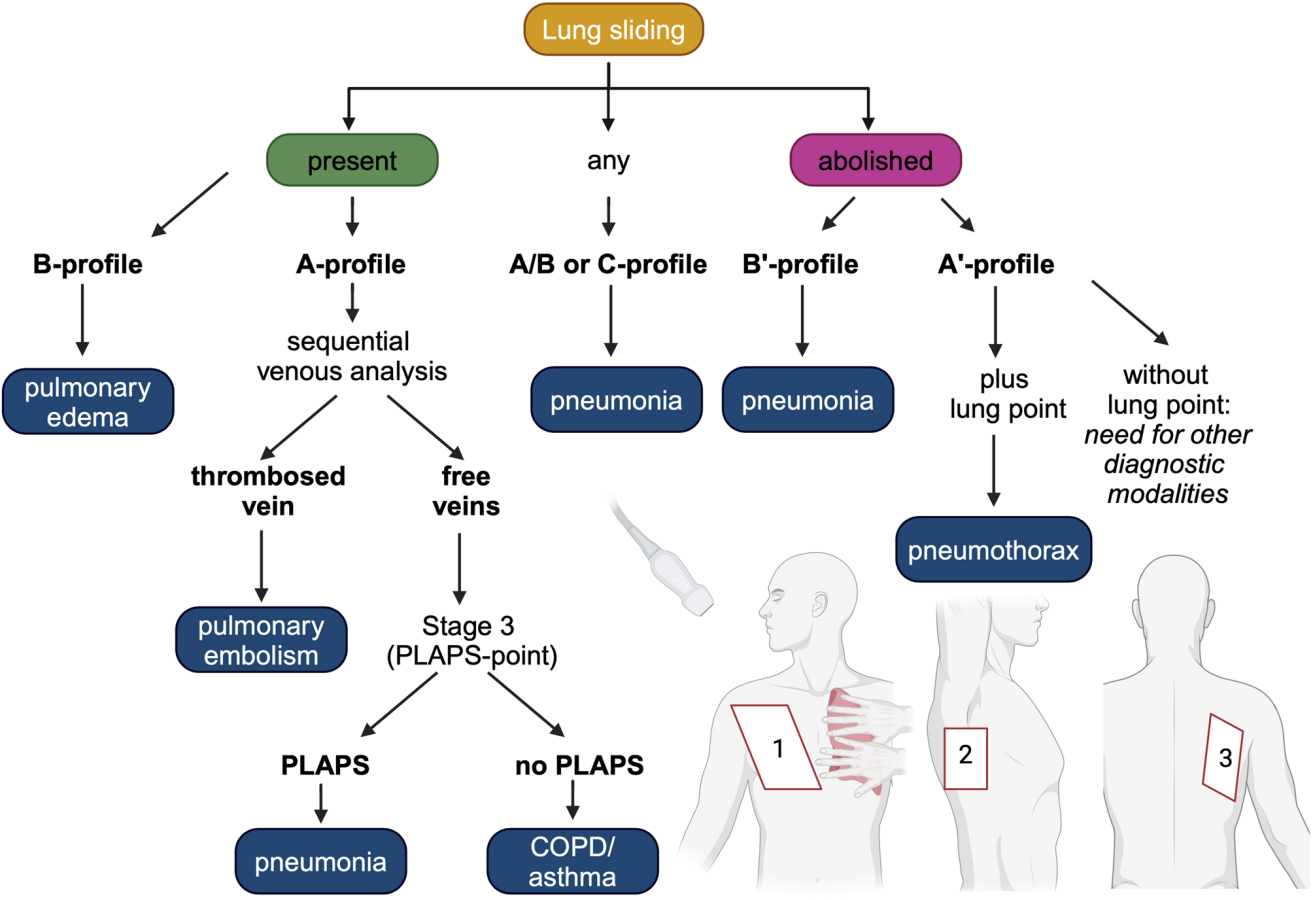
The BLUE protocol. The BLUE protocol involves sonographic assessment of three areas using two hands the size of the patient’s own to project the lung onto the thoracic wall [40]. These three zones are each divided in upper and lower halves. Physiologically, the lung displays lung sliding and A-lines. In the BLUE protocol, signs are combined resulting in seven profiles. Figure was adapted from Lichtenstein and colleagues [39, 41] and created with BioRender.com

Evidence suggests that prehospital interventions may reduce mortality, while misapplied therapies may be harmful [33, 34]making lung ultrasound a potentially useful tool with high sensitivity and specificity [35, 36]. One prominent example of structured PHUS is the Bilateral Lung Ultrasound in Emergency (BLUE) protocol developed by Lichtenstein and colleagues (Fig. 1) [35, 37]. In this protocol, three standardized fields on either side of the thorax are assessed for ten signs indicative of diagnoses associated with dyspnea [38]. The BLUE protocol has a diagnostic accuracy of > 90% and can be completed within 3 min [39].

The features shown in the BLUE protocol are briefly explained in the following:

#### Lung sliding

Lung sliding refers to the sliding of the visceral and parietal pleura that physiologically occurs during respiration [42]. This sign is not seen in conditions where the pleurae are not adherent, such as pneumothorax (air) or pleural effusion (fluid). Process research has shown that prehospital care providers can learn to detect the sliding lung sign easily with a high level of sensitivity (97%) and specificity (94%) and retain their skill in a 9-month follow-up [14].

#### A-lines

**Fig. 2:**
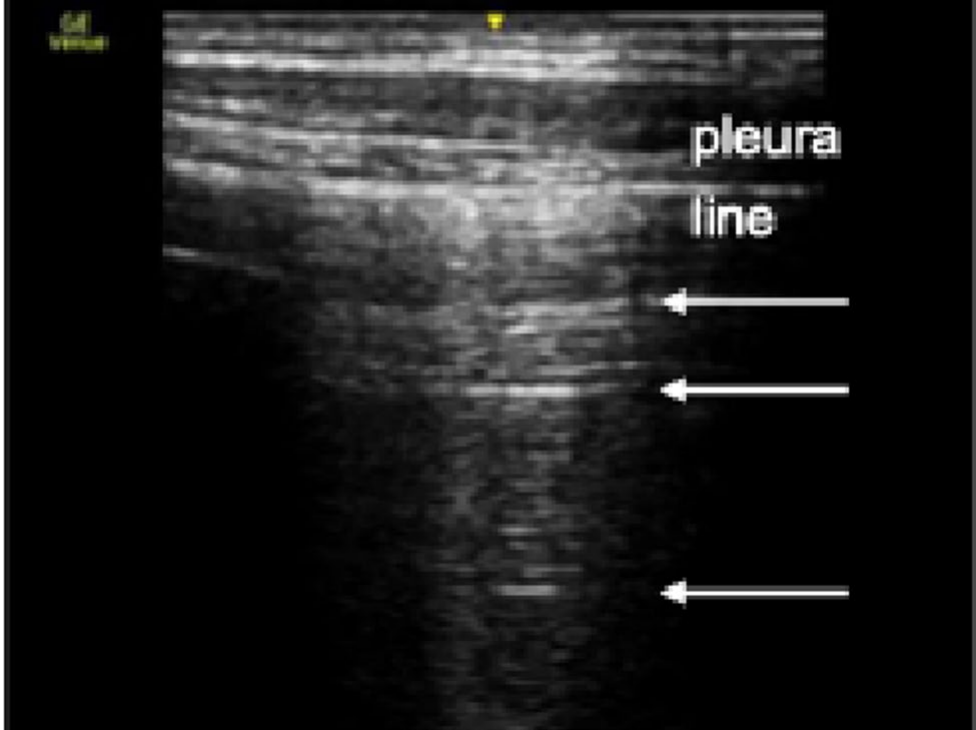
Ultrasound image of A-lines. Arrows indicate A-lines

Horizontal A-lines (Fig. 2) are artifacts that appear as echogenic reflections of the pleural line. Their presence results from gas below the parietal pleura, which reflects the ultrasound waves back to the transducer [43]. A-lines appear physiologically, but can still be present in cases of pneumothorax [43].

#### B-lines

**Fig. 3:**
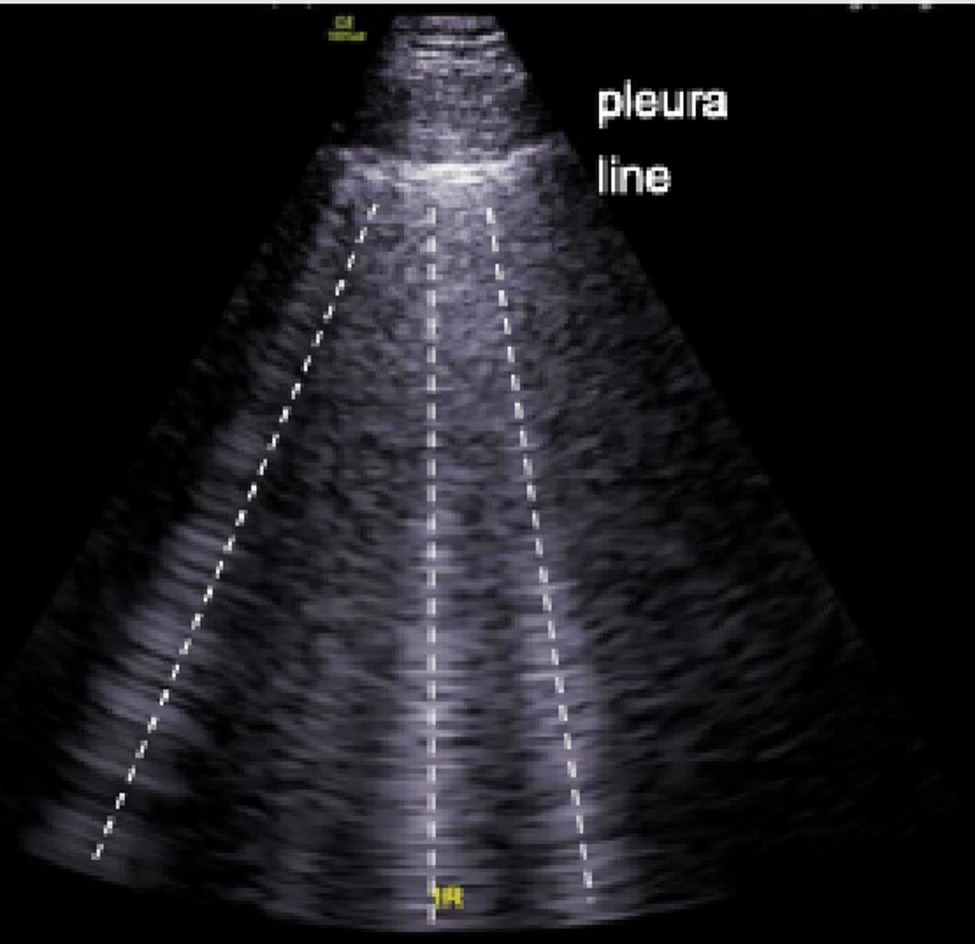
Ultrasound image of B-lines. Dashed lines represent “comet tail” artifact

B-lines (Fig. 3), also known as “lung comets”, represent accumulation of fluid in the pulmonary interstitium and appear as hyperechoic vertical reverberation artifacts originating from the pleural line [44].

For example, in patients with acute decompensated heart failure (ADHF), the number of B-lines correlates with X-ray signs of extravascular fluid [45, 46]. Hence, B-lines serve as a suitable indicator to differentiate between ADHF and COPD, a differential diagnosis which can be challenging in the prehospital environment [47]. A recent metaanalysis revealed ultrasound is a potent tool to distinguish between ADHF, pneumonia and exacerbation of COPD in general [48]. In a case-controlled study of patients with acute respiratory insufficiency, ultrasound was successfully used to distinguish between cardiac failure and exacerbation of obstructive lung disease in the prehospital setting [49]. In a pilot study assessing PHUS for the diagnosis of cardiogenic pulmonary edema, ultrasound reached a sensitivity of 94,4% [50]. In prediction of high-altitude pulmonary edema, ultrasound proved to be a potent tool in early detection before symptoms appear [51]. Here, the number of B-lines was successfully used to guide treatment and may have similar applications for prehospital clinicians [52].

#### Posterolateral alveolar or pleural syndromes (PLAPS)

**Fig. 4:**
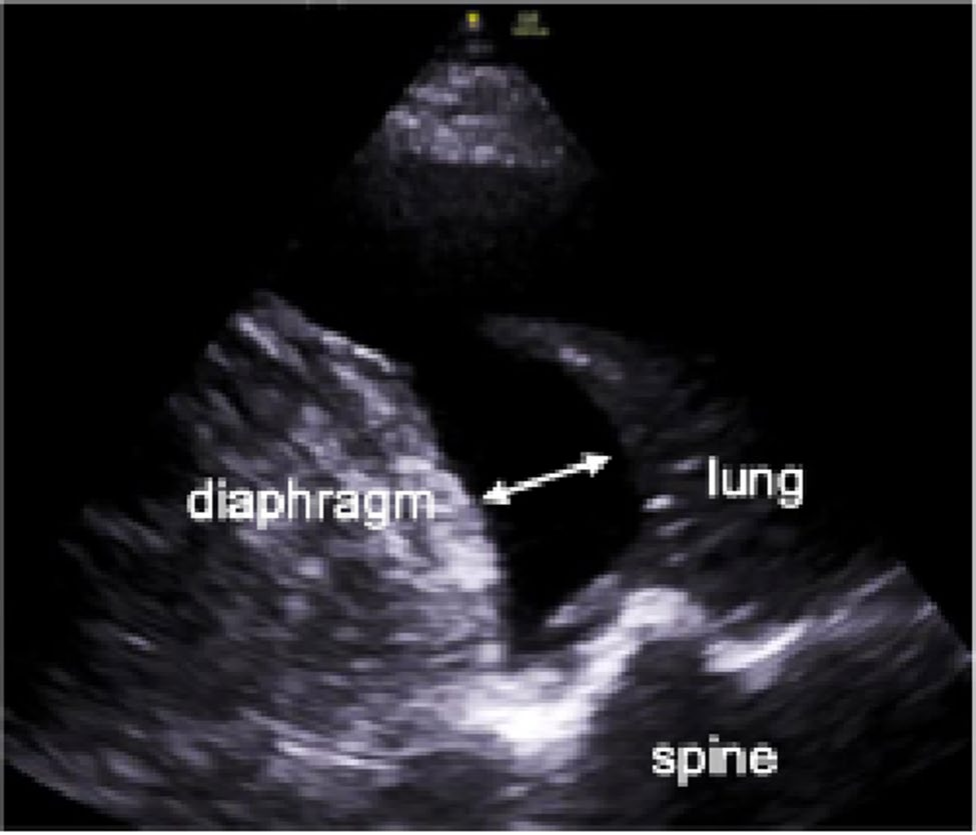
Ultrasound image of pleural effusion. Double arrow indicates largest distance between lung atelectasis and basal diaphragm

Pleural effusion (Fig. 4) may occur as a symptom of various diseases and can itself be the cause of respiratory distress. It can be detected at the PLAPS-point, a posterior area in supine patients where free effusions are located [38]. Importantly, ultrasound holds a far higher diagnostic accuracy for pleural effusion compared to bedside X-rays (93% vs. 47%) [53]. Even a small volume of 20 mL may become visible [54]. In a recent trial, 100% of congestive heart failure patients showed pleural effusion, while it was evident in only 20% of COPD patients [28].

#### Lung-point

**Fig. 5:**
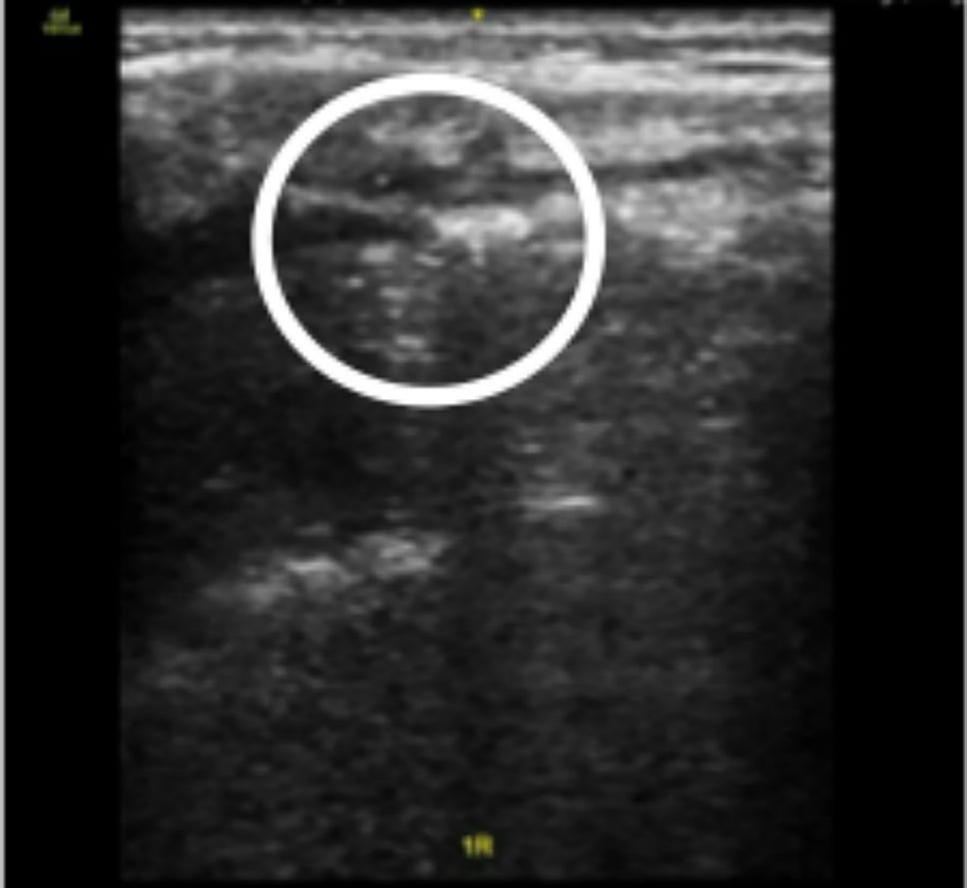
Ultrasound image of the lung-point (circled) of a patient presenting with pneumothorax

The “lung-point” (Fig. 5) is pathognomonic for pneumothorax [55]. In patients with an A’-profile, B-lines and lung-sliding suddenly appear during respiration due to the inspiratory increase of parietal contact of the collapsed lung.

#### Transthoracic echocardiography (TTE)

**Fig. 6:**
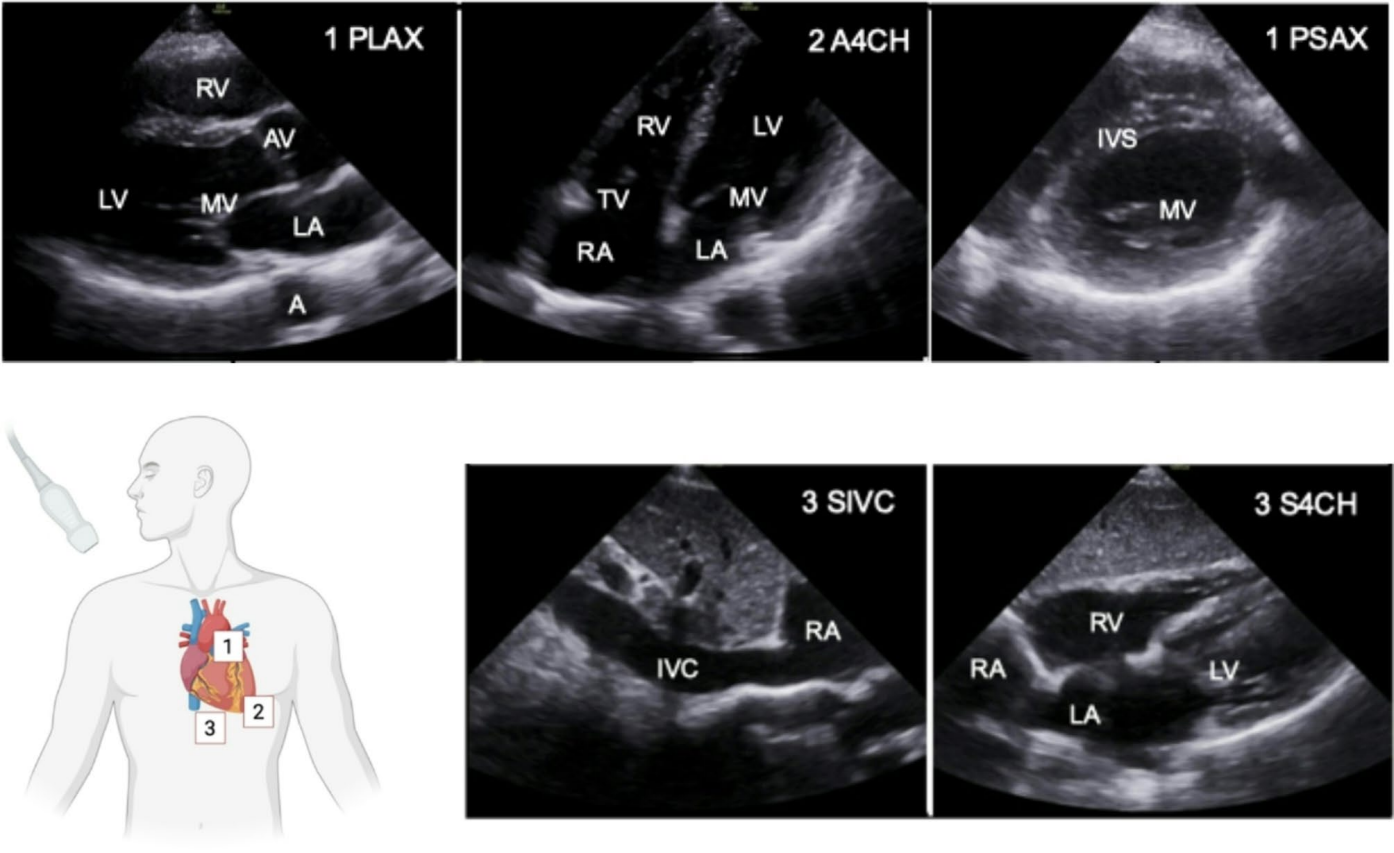
Basic FoCUS examination views. Prehospital TTE can be performed using the parasternal window (**1**) to assess the parasternal long-axis (PLAX) and short-axis (PSAX), the apical 4-chamber-view (A4CH) (**2**) and the subcostal window (**3**) for the subcostal inferior vena cava (SIVC) and subcostal 4-chamber (S4CH) [56]. A = aorta, AV = aortic valve, IVC = inferior vena cava, IVS = interventricular septum, LA = left atrium, LV = left ventricle, MV = mitral valve, RA = right atrium, RV = right ventricle, TV = tricuspid valve. Created with BioRender.com

In addition to lung thoracic ultrasound, TTE offers the experienced investigator another modality to distinguish between cardiac and pulmonary etiologies. Here, Focus cardiac ultrasound (FoCUS) (Fig. 6) is a standardized examination to diagnose or rule out hallmarks of relevant cardiac pathologies [56]. Recently, prehospital TTE was identified as a suitable means for early identification of regional wall motion abnormalities, if NSTEMI was suspected [57]. In a case series, PHUS successfully identified evidence of massive pulmonary embolism in hemodynamically unstable patients, pointing towards an application to aid in thrombolysis indication and timing [58].

### Respiratory distress– ultrasound-guided interventions

#### Tube thoracostomy

Characteristic sonographic signs of pneumothorax include the appearance of A-lines, absence of B-lines and absence of lung sliding [37]. In a previous study, ultrasound changed therapeutic management in 21% of cases, including aborting tube thoracostomy in 4% of cases and changing hospital destination in 4% of cases [59]. Currently, up to 25% of tube thoracostomies are associated with complications, which include injury to underlying organs, hemothorax or unnecessarily created thoracostomies, when performed without sonography in the prehospital setting [60]. With the high complication rate associated with tube thoracostomies, PHUS may aid in avoiding low insertion, arterial, and visceral injuries.

#### Intubation

First-attempt success rates of prehospital intubations vary widely between 46% and 85% [61]. Use of sonography to identify correct endotracheal tube placement was first described in neonates in 1986 [62]. In emergency intubations, transtracheal ultrasound was found to be 98% sensitive and 94% specific [63]. Although capnography is still the gold standard confirming correct tube position, it does not rule out endobronchial intubation [64]. In a prehospital setting, Zadel and colleagues confirmed endotracheal tube position by sonographic verification of bilateral lung sliding and diaphragmatic excursion. Esophageal intubation was only detected in 30% of cases visually or by auscultation, while sensitivity and specificity of PHUS were 100% [65]. The performance of PHUS for verification of tube position took a median time of 30 s (8–120 s). The ultrasound-guided tube position verification was also transferable to a pediatric setting [66]. PHUS may thus be a suitable additional device for further verification of tube position.

#### Difficult airway

In preclinical studies assessing cricothyroidotomy with human cadavers with difficult or impossible landmark palpation, use of ultrasound significantly decreased the incidence of laryngotracheal injuries, while increasing the probability of correct insertion by 5.6 times [67]. Further, a novel technique of bougie-assisted cricothyroidotomy guided by ultrasound was described previously [68]. However, use of ultrasound in cricothyroidotomy is still under debate. In case of blunt laryngeal trauma, airway management may particularly be challenging [69]. In the rare but life-threatening case of laryngotracheal separation, dyspnea may be mistaken for a symptom of swelling or bleeding, resulting in intubation attempts with misplacement of the endotracheal tube. A previously published review proposes PHUS as an important tool for assessment of blunt laryngeal trauma to identify laryngotracheal separation [70].

#### Continuous positive airway pressure (CPAP)

PHUS may be used to monitor the effectiveness of prehospital CPAP treatment in ADHF. In a recent study, use of prehospital CPAP decreased B-lines and improved respiratory and hemodynamic variables [71].

### Circulation

#### Cardiac arrest– diagnostics

**Fig. 7:**
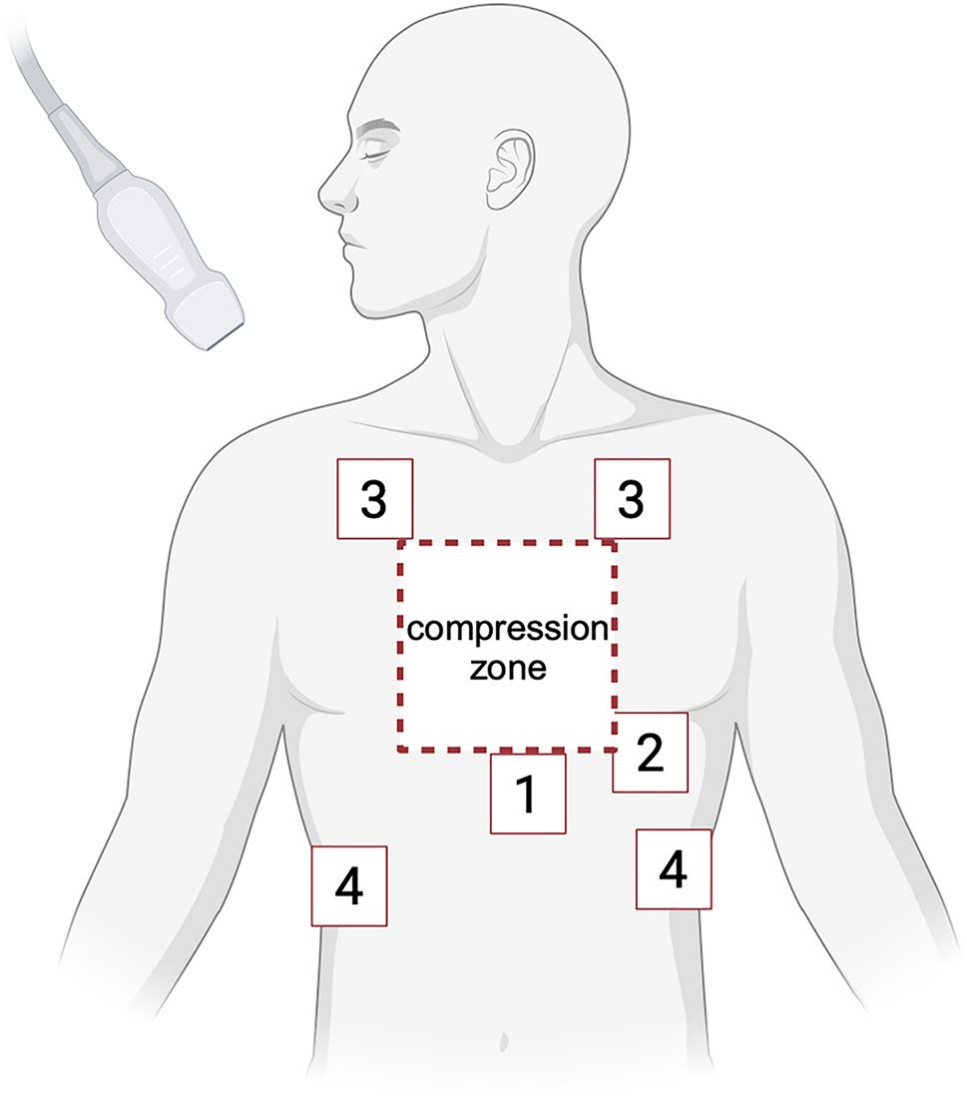
Ultrasound probe positioning during chest compressions. (**1**) subcostal window and (**2**) apical 4-chamber-view, (**3**) pulmonary view and (**4**) Morison’s with hemothorax or splenorenal with hemothorax respectively, can be used to assess reversible causes. Adapted from Ávila-Reyes et al. [73] and created with BioRender.com

The current ERC guidelines recommend the use of point-of-care ultrasound to detect reversible causes of cardiac arrest [72]. In particular, hypovolemia, cardiac tamponade, tension pneumothorax and pulmonary thrombosis can be evaluated as reversible causes using ultrasound (Fig. 7). It is emphasized, however, that ultrasound diagnostics during cardiac arrest require a skilled operator and that the highest priority lies in minimizing interruptions of chest compressions. Ultrasound should be prepared during CPR and can be performed during rhythm and pulse check and importantly, should not impede compression pauses. Subcostal window (1) and apical 4-chamber-view (2) can be used to assess cardiac motion, rule out pericardial effusion or right heart strain. In addition, pulmonary window (3) may rule out tension pneumothorax, while the abdominal views (4) may exclude intraabdominal fluid. Of note, the different views may have to be performed during different compression pauses.

In a FoCUS study by Breitkreuz et al., ultrasound examinations were performed in compliance with current guidelines and did not interfere with uninterrupted high-quality CPR [27]. In contrast, there are contradictory studies pointing towards ultrasound leading to prolonged resuscitation pauses during cardiac arrest [74, 75]. Again, ultrasound undoubtedly requires a skilled examiner to offer diagnostic benefits with the priority of minimum compression pauses.

Interestingly, in patients with suspected pulseless electrical activity (PEA), FoCUS showed coordinated cardiac motion (pseudo-PEA) in 75% of cases indicative of increased survival [27]. Conversely, survival of pulseless traumatic arrest without sonographic cardiac activity was rare with no ROSC after a standstill of 10 min [27, 76]. Hence, ultrasound may provide another dimension to prehospital advanced life support. However, the current guidelines do not recommend terminating CPR solely based on sonography [72].

#### Cardiac arrest– ultrasound-guided interventions

Sonography-guided interventions may be employed during non-traumatic and traumatic cardiac arrest. In an unselected study population of 230 patients with cardiac arrest of any etiology, Breitkreuz et al. found that treatable causes of cardiac arrest were reduced ventricular function (59%), pericardial tamponade (9.8%), right ventricular dilation (7.8%) and hypovolemia (3.9%) [27]. In this study, the ultrasound findings altered therapy in 66–89% of cases. New therapy modalities included pericardiocentesis, fluid administration or inotropic therapy. Moreover, ultrasound findings significantly influenced the choice of destination hospital. In addition, ultrasound may be used to determine ventricular capture in transcutaneous pacing [77].

### Hemodynamics– diagnostics

#### Nontraumatic shock

**Fig. 8:**
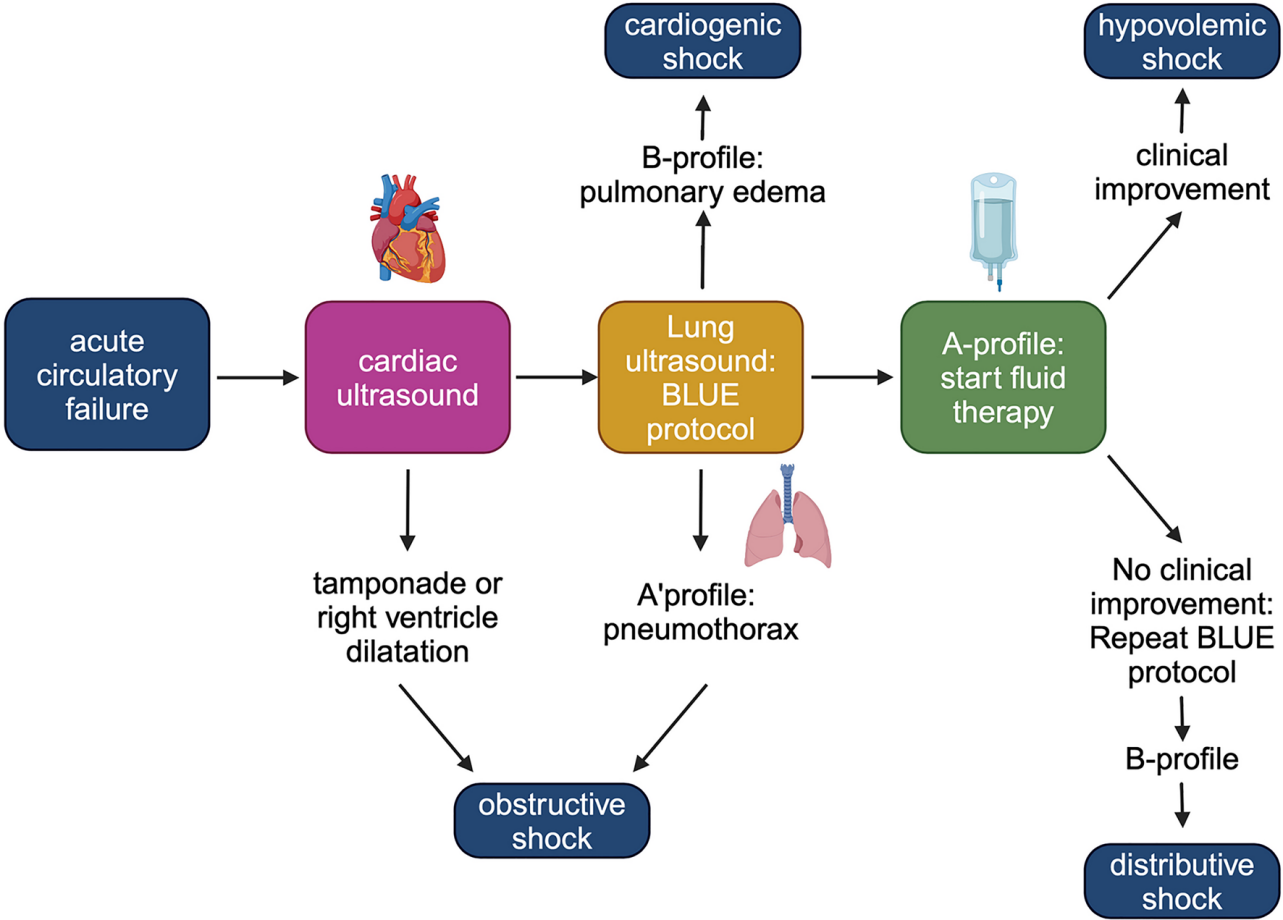
The FALLS protocol. Decision tree for the management of unexplained shock with the use of cardiac and lung ultrasound. Adapted from Lichtenstein et al. [37] and created with BioRender.com

The FALLS (Fluid Administration Limited by Lung Sonography) protocol (Fig. 8) is a dynamic imaging modality for the differential diagnosis of shock, aiming to provide early fluid therapy, particularly in septic shock. It includes the BLUE protocol mentioned earlier in this review (Fig. 1). Application of the FALLS protocol takes a total duration of approximately 5 min for the initial assessment by the skilled operator [41].

**Fig. 9:**
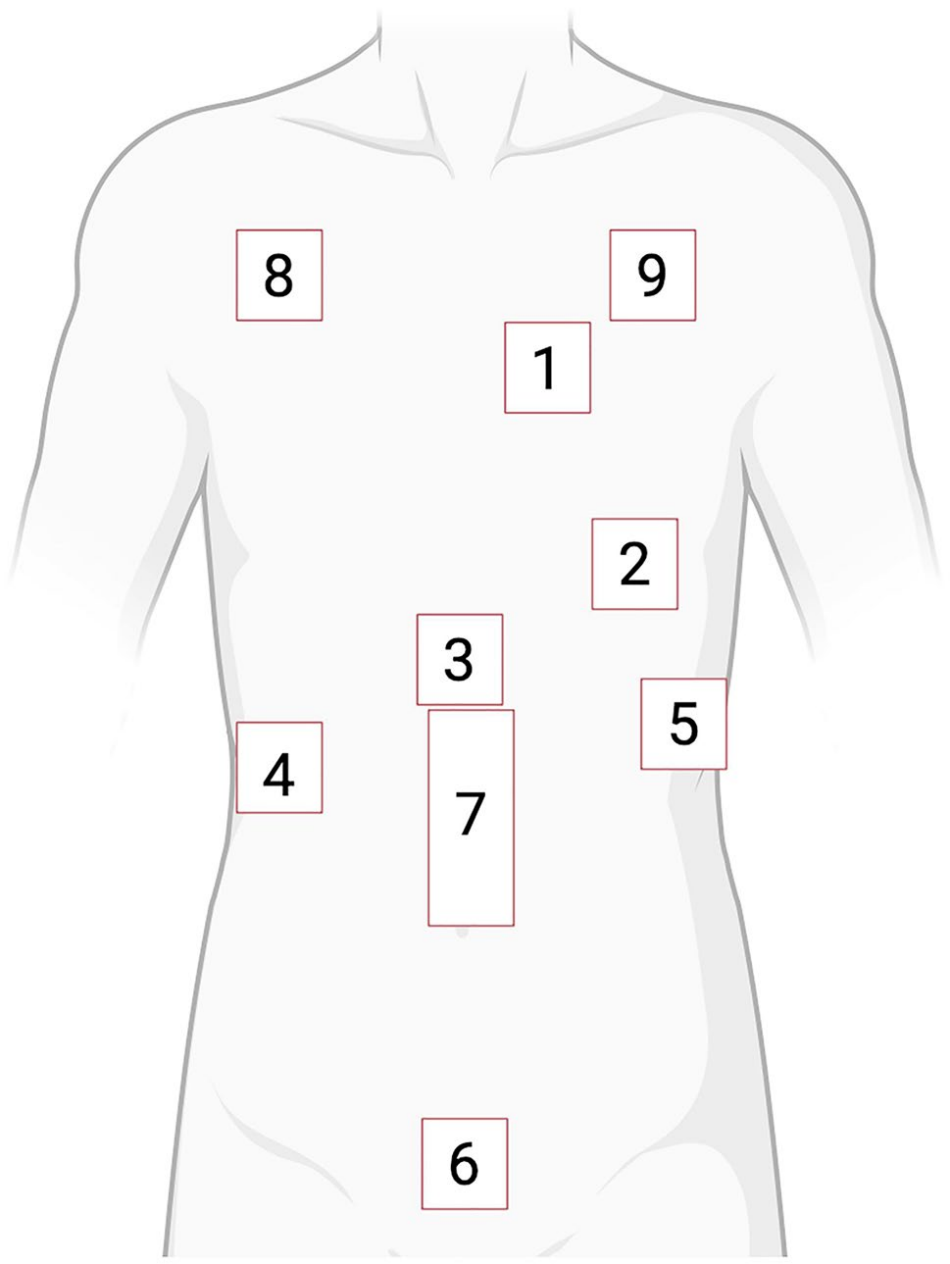
Probe positioning in the RUSH exam. (**1**) parasternal long and (**2**) apical four-chamber view assess the “pump” to evaluate pericardial effusion and ventricular function. (**3**) inferior vena cava, (**4**) Morison’s with hemothorax, (**5**) splenorenal with hemothorax, (**6**) bladder view asses the “tank”, aiming to dissect volume status of the vena cava and evidence for pleural effusion or hemoperitoneum. (**7**) aortic slide view evaluates aortic aneurysm or dissection, which may be supplemented by evaluation of deep vein thrombosis (the “pipes”). (**8**,** 9**) pulmonary views are used for rapid detection of pneumothorax. Adapted from Perera et al. [78] and created with BioRender.com

The Rapid Ultrasound in SHock (RUSH, Fig. 9) exam published by Perera and colleagues is another protocol of sonographic evaluation of shock [78].

### Trauma– diagnostics

#### eFAST

**Fig. 10:**
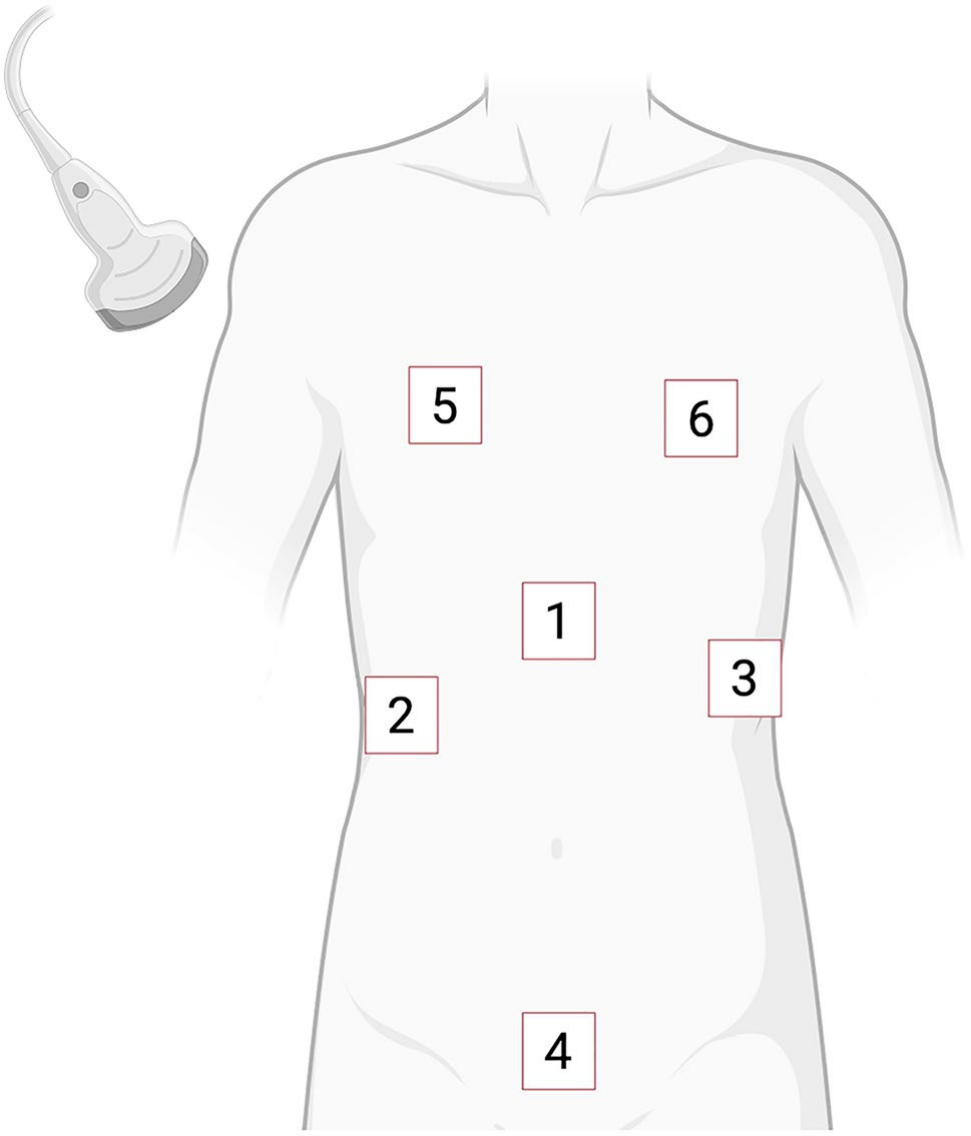
Ultrasound probe position for eFAST. eFAST can be used assessing the pericardium (**1**) for pericardial effusion, right (**2**) and left (**3**) upper quadrant for respective peritoneal or pleural fluid, suprapubic area (**4**) for peritoneal fluid and right (**5**) and left (**6**) anterior thoracic region for pneumothorax [83]. Created with BioRender.com

eFAST (Fig. 10) is the gold standard point-of-care protocol for diagnosing or ruling out pneumothorax, pericardial effusion and abdominal free fluid in trauma patients [79]. In different surveys, eFAST was identified as the most commonly used application of PHUS reaching comparable sensitivity and specificity between in-hospital and prehospital settings [80]. In a 2024 meta-analysis, the specificity of detection of free intraabdominal fluid by PHUS reached 97% and significantly reduced time to hospital and operative treatment [81]. As false-negative results occur, especially when FAST is performed in the early post-injury phase, repetition of FAST is recommended, leading to a 50% reduction in false negatives [82]. While eFAST is an important tool for rapid identification of intraabdominal free fluid and other traumatic injuries, it may not detect retroperitoneal hemorrhage.

#### Abdominal organ injuries

Importantly, eFAST may not detect solid organ injuries. Conversely, detection of traumatic pneumoperitoneum due to gastrointestinal perforation by sonography can be a useful tool, in which the experienced examiner reaches 85–90% sensitivity and 100% specificity [82]. Other injury patterns such as diaphragmatic rupture occur in 5% of blunt abdominal traumas and may present despite negative FAST. While eFAST offers a rapid way for clinicians to diagnose many life-threatening traumatic injuries, the exam is not without limitations. Prehospital clinicians should understand its utility for ruling in pathologies, while recognizing its limitations. Compared to diagnostic laparocopy as a gold standard, FAST sonography only reached a sensitivity of 50% in detecting diaphragmatic rupture [84]. Poor diaphragmatic movement or elevation, liver sliding sign, subphrenic effusion, presence of intrathoracic spleen or liver are indicative of diaphragmatic rupture [85]. Evidence of sonographic detection of diaphragmatic rupture is currently based on several case reports from intrahospital point-of-care ultrasound [85–88]which merits extrapolation to the prehospital setting in future studies and importantly, requires a skilled operator.

#### Fractures

Traditionally in military and remote locations, ultrasound has been used to detect long bone extremity fractures [89, 90]. In a military station, ultrasound altered the disposition of patients and held 100% sensitivity and 94% specificity for fracture detection, when performed by board-certified emergency medicine physicians [91]. Subsequently, ultrasound may be helpful in areas where radiography service is unavailable.

### Trauma– ultrasound-guided interventions

#### Hemodynamics

Ultrasound-guided catheter placement is an attractive method in patients with difficult intravenous access [92]. Recently, a pilot study successfully evaluated the implementation of PHUS to gain peripheral intravenenous access in out-of-hospital patients with predicted difficult venous access by trained paramedics [93]. Of note, if intravenous access is not available in unstable patients, intraosseous access should be established without delay, as recommended in the current ERC guidelines [72]. Further, continuous ultrasound-guided visualization is an effective and safe measure for pericardiocentesis [94]. In 2014, London’s Air Ambulance Charity’s trauma team performed the first ultrasound-guided resuscitative endovascular balloon occlusion of the aorta (REBOA), as an “internal” aortic cross-clamping in pelvic fracture [95]. As current evidence of ultrasound-guided REBOA is mainly based on case reports [96] and animal studies [97, 98]further research on proper indications and its feasibility in prehospital care is needed. Provision of prehospital extra corporeal life support (ECLS) has been implanted in some regions [99, 100]. Most likely, PHUS may assist the experienced clinician in ECLS cannula placement.

#### Regional anesthesia

In a randomized controlled trial, use of PHUS-guided nerve blocks significantly reduced pain intensity and severity compared to commonly used systemic analgesia in trauma patients [101]. Here, ultrasound-guided femoral nerve block may be a method to reduce pain in patients with hip fractures [102]. Further, trained emergency medical nurses were able to successfully perform prehospital fascia iliaca compartment block in patients with suspected proximal femur fracture [103]. Additional types of regional anesthesia were successfully tested in challenging environments [104].

### Other diagnostic and therapeutic applications

#### Stroke

Reducing time between symptom onset of ischemic stroke and thrombolysis is the utmost priority to save the penumbra, and thereby reducing mortality and improving neurological outcome [105]. A recent analysis found that only one fourth of patients arrive in hospital within the time window for lysis therapy [106]. Hence, rapid on-scene detection may accelerate patient disposition. Transcranial sonography in the proximal M-1 segment was identified to yield 90% sensitivity in diagnosis of middle cerebral artery occlusion [107]. Obtaining ultrasound images through the temporal window can be challenging and thus requires profound expertise and may not be suitable for widespread use. Regarding ultrasound as a therapeutic application, sonothrombolysis in acute ischemic stroke was recently tested in a randomized multicenter trial, but stopped due to lack of clinical benefit [108]. Further, intrahospital imaging modalities are still the diagnostic gold standard to justify lysis and other therapeutic interventions [109]. Hence, the current benefit of prehospital ultrasound in stroke may particularly consist of accelerated triage to an appropriate hospital.

#### Intracranial pressure

Optic nerve sheath diameter (ONSD) is a measure to identify increased intracranial pressure (ICP). In a small group of 35 patients, ONSD measurements had a 100% sensitivity and 95% specificity compared to CT scan in predicting elevated ICPs [110]. Like PHUS in stroke, ONSD measurements may be a suitable screening tool to start neuroprotective strategies on-scene or during transport and to assist in proper hospital allocation.

#### Obstetrics

Assessing fetal health during transport to a high-risk obstetrical unit may be challenging with traditional monitoring. In a series of case reports, the fetal evaluation for transport with ultrasound (FETUS) protocol was recognized as suitable to determine fetal heart rate, position, movement, and condition of the placenta during aeromedical transport [111]. In another case report, PHUS was successfully used to diagnose a ruptured ectopic pregnancy [112].

### Future perspectives– the role of artificial intelligence-assisted ultrasound interpretation

Artificial Intelligence (AI) is increasingly implemented into several fields of medicine, especially imaging. Traditionally, AI research has focused on radiographic sectional imaging. These images often offer a more standardized view than dynamic imaging, such as ultrasound. However, as the recommended views for ultrasound assessment are becoming more standardized, this opens up opportunities for AI to be trained on sufficient amounts of data to make accurate predictions. In a recent observational study, a neural-network architecture was used for automated detection of pneumothorax with ultrasound. The model achieved a good sensitivity of 86% with a specificity of 75%, despite being trained on a small number of images (*n* = 30). However, this is not yet on par with human-level interpretation, which achieves much higher sensitivity and specificity, as described above. Another study assessed detection of B-lines by AI compared to experts. Again, AI was not able to match human-level performance, at least at the expert level here as well [113]. In a direct comparison between the Auto-B-Lines algorithm (General Electric Healthcare) and expert-level ultrasound, both, the human expert and the AI achieved high levels of sensitivity (96.7% vs. 95.6%). However, the human expert achieved noticeably higher specificity (79.1% vs. 64.1%) [114. The development of new models requires a much larger dataset, but public datasets of ultrasound are currently lacking. The publication of such datasets in other fields, such as the interpretation of x-ray images, has brought an evolution of AI models in the past that were able to reliably predict pathologies and constantly improve performance [115]. This represents an opportunity for the medical community to work together on creating such ultrasound datasets to improve model development in the future. At the current stage of evicende, however, AI cannot replace clinical decision making and may only serve as an adjunct tool.

## Conclusions

**Fig. 11:**
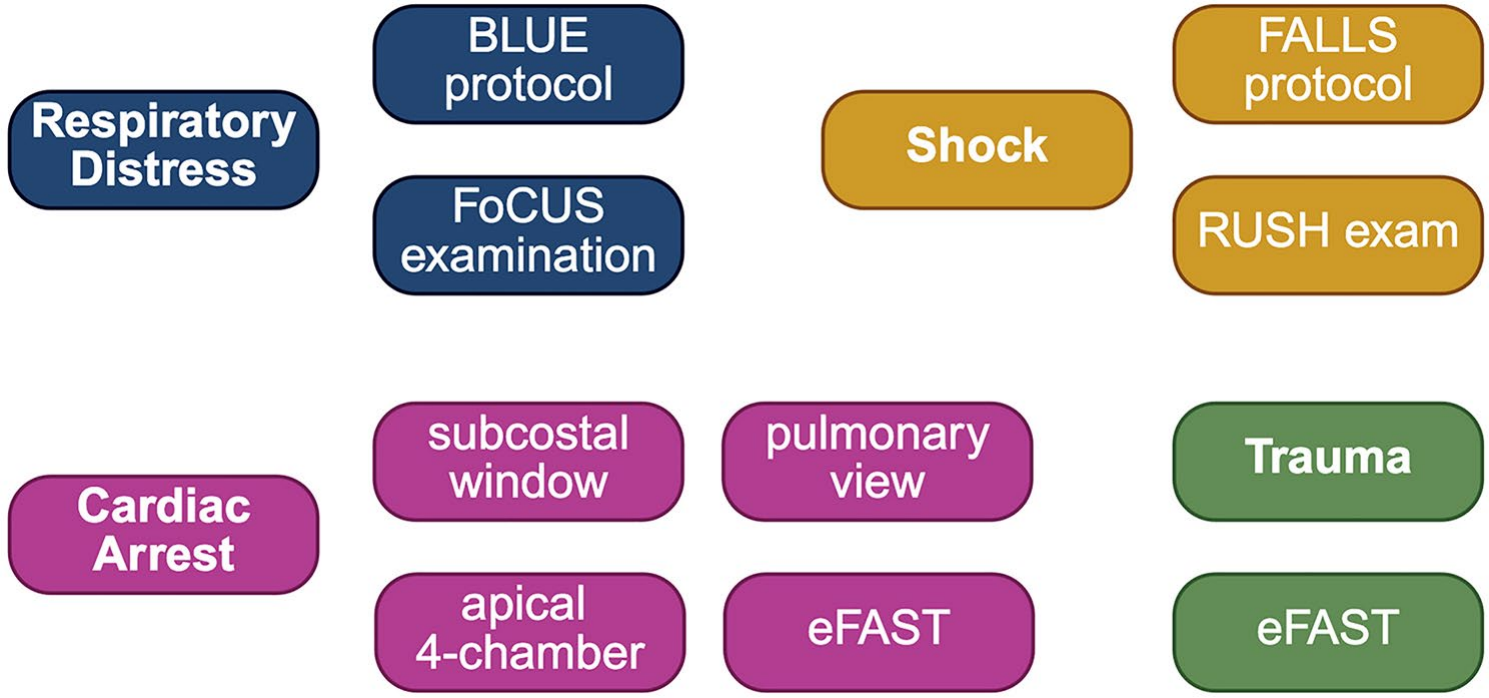
Point-of-care sonography applications in major prehospital symptom complexes

PHUS is an emerging frontline diagnostic and therapeutic tool with the potential to advance prehospital emergency medicine. Different protocols are available that may guide the well-trained practicioner in major prehospital symptom complexes, including respiratory distress, cardiac arrest, shock and trauma (Fig. 11; Table 2). Rapid assessment by PHUS may guide treatment decisions and improve triage to appropriate hospitals (Table 3). In this regard, early diagnosis may facilitate the triage to specialty centers, for example to cardiac centers, if pericardial effusion is detected, or to trauma centers, if internal bleeding is visualized by FAST sonography or stroke centers, if stroke is suspected in transcranial doppler. Further, in an era of telemedicine, images may be transmitted to respective destination hospitals. To avoid the risk of prolonging preclinical procedural times, the use of ultrasound must be stringently integrated into distinct diagnostic and therapeutic algorithms. Importantly, the speed of technological advances requires curricula that define a cohesive framework for educational and training programs, resulting in competency and maintenance of operator skills. The differentiated use of PHUS and the associated training concepts further emphasize the need for increasing professionalization in prehospital emergency medicine. As prehospital care is often mistaken for a one-size-fits-all therapeutic field, constant advances in PHUS may significantly improve the field, moving it closer to provision of personalized medicine. There is growing evidence supporting the use of PHUS, however, to date, few systematic data exist and evidence for management of certain disease patterns is currently soley based on case reports. The current lack of randomized controlled trials will hopefully soon translate into large scale clinical trials to further elucidate the real clinical impact of PHUS.

**Table 2:** Diagnostic accuracy of key point-of-care sonography protocols

Protocol	Disease pattern	Sensitivity	Specificity
Blue protocol [41]	Acute hemodynamic pulmonary edema	97%	95%
	COPD in exacerbation or severe acute asthma	89%	97%
	Pulmonary embolism	81%	99%
	Pneumothorax	88%	100%
	Pneumonia	89%	94%
eFAST [79]	Pneumothorax	69%	99%
	Pericardial effusion	91%	94%
	Intra-abdominal free fluid	74%	98%
FoCUS [116]	Left ventricular dysfunction	84%	89%
RUSH exam [117]	Hypovolemic shock	100%	94%
	Cardiogenic shock	89%	97%
	Obstructive shock	94%	98%
	Distributive shock	73%	100%

**Table 3:** Therapeutic consequences of PHUS. (adapted from Vianen et al. [4])

Patient destination change	7,7%
Drug therapy change	12,9%
Fluid therapy change	9,1%
Invasive procedure performed/called off	2,9%
CPR initiated/terminated	13,4%
Evaluation of treatment effect	10,5%

## Data Availability

No datasets were generated or analysed during the current study.

## Abbreviations

A: Aorta
A4CH: Apical 4-Chamber-View
ADHF: Acute Decompensated Heart Failure
AI: Artificial Intelligence
AV: Aortic Valve
BLUE: Bilateral Lung Ultrasound in Emergency
COPD: Chronic Obstructive Pulmonary Disease
CPAP: Continuous Positive Airway Pressure
CPR: Cardiopulmonary Resuscitation
CT: Computed Tomography
ECLS: Extra Corporal Life Support
eFAST: Extended Focused Assessment with Sonography for Trauma
ERC: European Research Council
FALLS: Fluid Administration Limited by Lung Sonography
FAST: Focused Assessment with Sonography for Trauma
FETUS: Fetal Evaluation for Transport with Ultrasound
FoCUS: Focus Cardiac Ultrasound
ICP: Intracranial Pressure
IVC: Inferior Vena Cava
IVS: Interventricular Septum
LA: Left Atrium
LV: Left Ventricle
MV: Mitral Valve
ONSD: Optic Nerve Sheath Diameter
PEA: Pulseless Electoral Activity
PHUS: Prehospital Point-of-Care Ultrasound
PLAPS: Posterolateral Alveolar or Pleural Syndromes
PLAX: Parasternal Long-Axis
PSAX: Parasternal Short-Axis
RA: Right Atrium
REBOA: Resuscitative Endovascular Balloon Occlusion of the Aorta
RUSH: Rapid Ultrasound in Shock
RV: Right Ventricle
S4CH: Subcostal 4-Chamber
SIVC: Subcostal Inferior Vena Cava
TTE: Transthoracic Echocardiography
TV: Tricuspid Valve
